# Survival outcomes for lung neuroendocrine tumors in California differ by sociodemographic factors

**DOI:** 10.1530/ERC-23-0068

**Published:** 2023-12-08

**Authors:** Claire K Mulvey, Alan Paciorek, Farhana Moon, Paige Steiding, Brandon Shih, Matthew A Gubens, Li Zhang, Emily K Bergsland, Iona Cheng

**Affiliations:** 1 Helen Diller Family Comprehensive Cancer Center, University of California, San Francisco, California, USA; 2 Division of Hematology/Oncology, Department of Medicine, University of California, San Francisco, California, USA; 3 Department of Epidemiology and Biostatistics, University of California, San Francisco, California, USA

**Keywords:** typical carcinoid, atypical carcinoid, bronchial carcinoids, pulmonary neuroendocrine tumors

## Abstract

Lung neuroendocrine tumors (NETs) have few known predictors of survival. We investigated associations of sociodemographic, clinicopathologic, and treatment factors with overall survival (OS) and lung cancer-specific survival (LCSS) for incident lung NET cases (typical or atypical histology) in the California Cancer Registry (CCR) from 1992 to 2019. OS was estimated with the Kaplan–Meier method and compared by sociodemographic and disease factors univariately with the log-rank test. We used sequential Cox proportional hazards regression for multivariable OS analysis. LCSS was estimated using Fine-Gray competing risks regression. There were 6038 lung NET diagnoses (5569 typical, 469 atypical carcinoid); most were women (70%) and non-Hispanic White (73%). In our multivariable model, sociodemographic factors were independently associated with OS, with better survival for women (hazard ratio (HR) 0.62, 95% confidence interval (CI) 0.57–0.68, *P* < 0.001), married (HR 0.76, 95% CI 0.70–0.84, *P* < 0.001), and residents of high socioeconomic status (SES) neighborhoods (HR_Q5vsQ1_ 0.73, 95% CI 0.62–0.85, *P* < 0.001). Compared to cases with private insurance, OS was worse for cases with Medicare (HR 1.24, 95% CI 1.10–1.40, *P* < 0.001) or Medicaid/other public insurance (HR 1.45, 95% CI 1.24–1.68, *P* < 0.001). In our univariate model, non-Hispanic Black Californians had worse OS than other racial/ethnic groups, but differences attenuated after adjusting for stage at diagnosis. In our LCSS models, we found similar associations between sex and marital status on survival, but no differences in outcomes by SES or insurance. By race/ethnicity, American Indian cases had worse LCSS. In summary, beyond disease-related and treatment variables, sociodemographic factors were independently associated with survival in lung NETs.

## Introduction

Neuroendocrine neoplasms (NENs) are a heterogeneous group of cancers that arise from neuroendocrine cells in almost any organ in the body. NENs can produce hormones and are classified histologically as well-differentiated neuroendocrine tumors (NETs) or poorly differentiated neuroendocrine carcinomas (NECs). Well-differentiated NETs of the lung account for less than 3% of all primary lung cancers in adults (Rekhtman 2010). However, the lung is the second most common NET primary site after the gastrointestinal tract (Modlin *et al.* 2003). Lung NETs have a wide spectrum of clinical behavior and a unique pathologic classification as either typical carcinoids (grade 1) or atypical carcinoids (grade 2) (Travis *et al.* 2015).

There are few established predictors of mortality for lung NETs beyond disease-specific factors like stage and grade (García-Yuste *et al.* 2007, Steuer *et al.* 2015). One recent publication found that sociodemographic factors were associated with survival for patients with lung NENs in the United States, including sex, marital status, insurance, race, and county of residence – with better survival noted for women, married, insured, Hispanic and ‘other’ race, and urban populations (Shah *et al.* 2021). However, this analysis did not account for treatment factors or socioeconomic status (SES) of cases, which may be important mediators of survival outcomes. In addition, the analysis included both lung NETs and NECs (small cell and large cell) in the same survival models. Given that well-differentiated lung NETs have different biology, genomic profiles, underlying risk factors, and treatment strategies than high-grade pulmonary NECs (Fernandez-Cuesta *et al.* 2014, Rekhtman 2022), we aimed to examine predictors of survival for well-differentiated lung NETs, specifically.

Using the California Cancer Registry (CCR), we characterized the clinical characteristics and survival outcomes of patients diagnosed with well-differentiated lung NETs in California from 1992 through 2019. In addition, we evaluated the impact of sociodemographic and treatment factors on survival.

## Materials and methods

### Data source

Analyses were performed using data from the CCR, part of the National Cancer Institute’s Surveillance, Epidemiology, and End Results (SEER) program. The CCR is the statewide population-based cancer registry in California. This study was approved by the Institutional Review Board of the University of California, San Francisco, and the California Committee for the Protection of Human Subjects. A waiver of informed consent was granted because all data were deidentified.

### Study population

The study population included all individuals aged 18 years and older in the CCR with an incident lung NET diagnosis from January 1992 through December 2019. We used tumor histology codes based on the *International Classification of Disease (ICD) for Oncology*, third edition (Fritz *et al.* 2000) and selected cases classified as either typical carcinoid (ICD-O-3 8240) or atypical carcinoid (ICD-O-3 8249) histology with an ICD-10 primary site code of the lung or bronchus (C34.0-34.9). Pulmonary atypical carcinoid cases were available beginning in 1998 due to the standardization of NET histology coding at that time (Travis *et al.* 1998). We excluded cases diagnosed at autopsy or via death certificate only. We also excluded individuals with poorly differentiated histologies, such as small- or large-cell NECs.

### Covariates

We obtained information routinely collected in the registry at the time of diagnosis including age, diagnosis year, sex, race/ethnicity (non-Hispanic White, non-Hispanic Black, Hispanic, Asian American/Pacific Islander, or American Indian), county of residence (rural, suburban, or urban), marital status (married or domestic partnered; or unmarried, including never married, separated, divorced, or windowed), tumor size, Charlson comorbidity index score, stage (localized, regional, or distant metastases using the SEER summary stage), and treatments within 12 months after diagnosis (surgery, chemotherapy, radiotherapy including peptide receptor radionuclide therapy, hormonal therapy including somatostatin analogs if used specifically to inhibit tumor growth, or immune therapies). Primary and secondary health insurance payer at diagnosis and/or the first year of treatment (no insurance/self-pay; private insurance only; any public, military, or Medicaid/Medi-Cal insurance; Medicare only or Medicare and private insurance) was collected for cases diagnosed after 1995, when insurance reporting was required in CCR. Patient address at diagnosis was geocoded and assigned to census block group to determine neighborhood level SES (nSES), created by principal component analysis of Census and American Community Survey data on education, housing, employment, occupation, income, and poverty (Yost *et al.* 2001, Yang *et al.* 2014).

### Outcomes

Our primary outcomes were overall survival (OS) and lung cancer-specific survival (LCSS). The CCR collects mortality information through active and passive follow-up and confirms cause of death using linkages to state and national vital statistics databases. Lung neuroendocrine cancer-specific death was determined from ICD-9 and ICD-10 topography codes and defined as death from malignant neoplasm of lung or bronchus, death from carcinoid syndrome, or, in the case of only one malignancy in the patient’s lifetime, death from endocrine neoplasms or neoplasms unspecified. The median (interquartile range) follow-up time for our lung NET cohort was 8.7 years (2.6-12.8).

### Statistical analysis

Patient characteristics at diagnosis were compared by stage using the Wilcoxon rank sum test for continuous variables and Pearson’s chi-squared test for categorical variables. We estimated OS by the Kaplan–Meier method, and the log-rank test was used to compare survival for each covariate of interest. We performed multivariable OS analyses using Cox proportional hazards regression models to measure the impact of sociodemographic predictors on survival, adjusting sequentially for an increasing number of covariates. Our models included previously published predictors, along with sociodemographic variables of interest, which were selected for final models based on statistically significant univariate results. Because the assumption of proportional hazards was violated for age, Cox models were age-stratified to allow baseline hazards to vary. Model 1 included sociodemographic and disease characteristics, including sex, race/ethnicity, county, marital status, nSES, stage, and decade of diagnosis. Model 2 included model 1 variables plus histology (typical or atypical carcinoid). Model 3 included model 2 variables plus treatment variables. Finally, model 4 included model 3 variables plus health insurance for the subset of patients diagnosed after 1995. Time of follow-up for analyses was from the date of diagnosis until death, date of last contact with the CCR, or end of the study period, whichever came first. For missing covariates, we used the missing indicator method for categorical variables in our multivariable models. For LCSS, we used the Fine-Gray subdistribution hazards regression models to estimate competing risks. Graphical examination of the cumulative incidence function curves by age levels did not suggest that proportionality assumption was violated, so age is included as a covariate in LCSS models. In secondary analyses, we included comorbidities as an additional covariate in models, and we generated additional fully adjusted models restricted to only those cases diagnosed after the year 1997 to account for the fact that atypical carcinoid histology was not standardized until 1998. Lastly, since prior published studies have not investigated the impact of nSES on survival in lung NETs, we examined whether there was heterogeneity in the effect of nSES on OS. We ran additional models that included interaction terms between nSES and other covariates. Interaction terms were tested by likelihood-ratio testing in nested models to assess for heterogeneity of effects. In instances of heterogeneity, stratum-specific hazard ratios (HRs) are reported separately. Statistical analyses were performed using STATA version 17.1 (Stata Corporation, College Station, TX). All *P* values were two-sided and *P* < 0.05 was considered statistically significant. No multiple testing adjustment was performed.

## Results

### Baseline characteristics of cases at diagnosis

Demographic and disease-related data are shown in Table 1 for all incident lung NET diagnoses in the CCR from 1992 through 2019 (*n* = 6038), the subset of cases who died from any cause during follow-up (*n* = 2183), and the subset of cases who died from lung NETs (*n* = 718). Most lung NET cases were women (70%), a majority identified as non-Hispanic White (73%), and cases tended to reside in areas of high nSES (25% in the highest statewide nSES quintile vs 11% in the lowest quintile).

**Table 1:** Demographic and clinical characteristics of the lung NET study population.

Variable	Level	Overall lung NET population (*n* = 6038)	Deaths (*n* = 2183)		Deaths from lung NET (*n* = 718)	
			*n*	% of total	*n*	% of total
Age at diagnosis	Median (IQR)	64 (53, 73)	70 (62, 77)		70 (60, 77)	
Diagnosis decade	1992–2000	1299	785	60.4	234	18.0
	2001–2009	1779	803	45.1	259	14.6
	2010–2019	2960	595	20.1	225	7.6
Sex^a^	Female	4213	1439	34.2	439	10.4
	Male	1824	743	59.3	279	15.3
Race/ethnicity^a^	NH White	4421	1689	38.2	538	12.2
	Hispanic	967	262	27.1	84	8.7
	NH Black	314	131	41.7	57	18.2
	Asian/Pacific islander	275	79	28.7	31	11.3
	American Indian	30	15	50.0	7	23.3
	Unknown	31	7	22.6	1	3.2
County	Urban	4311	1522	35.3	483	11.2
	Suburban	1567	607	38.7	219	14.0
	Rural	160	54	33.8	16	10.0
Marital status^a^	Unmarried	2436	986	40.5	323	13.3
	Married	3411	1134	33.2	372	10.9
	Unknown	191	63	33.0	23	12.0
Neighborhood SES^a^	Quintile 1 (lowest nSES)	672	263	39.1	81	12.1
	Quintile 2	1036	417	40.3	155	15.0
	Quintile 3	1354	506	37.4	188	13.9
	Quintile 4	1448	487	33.6	143	9.9
	Quintile 5 (highest nSES)	1527	509	33.3	151	9.9
Histology	Typical carcinoid	5569	2002	35.9	605	10.9
	Atypical carcinoid	469	181	38.6	113	24.1
Stage^a^	Localized	4008	1221	30.5	254	6.3
	Regional	1076	385	35.8	166	15.4
	Distant	739	445	60.2	248	33.6
	Unknown	215	132	61.4	50	23.3
Charlson comorbidities index^a^	None	2630	839	31.9	256	9.7
	1–2	2076	823	39.6	274	13.2
	≥3	484	256	52.9	81	16.7
	Unknown	848	265	31.3	107	12.6
Primary tumor size (cm)^a^	Median (IQR)	2.0 (1.3, 3.0)	2.0 (1.4, 3.0)		2.6 (1.5, 4.4)	
Surgery	Yes	4708	1406	29.9	359	7.6
	No	1330	777	58.4	359	27.0
Radiation^a,b^	Yes	260	190	73.1	124	47.7
	No	4946	1918	38.8	561	11.3
	Unknown	832	75	9.0	33	4.0
Chemotherapy^a^	Yes	311	200	64.3	135	43.4
	No	5688	1962	34.5	135	2.4
Hormonal therapy^a^	Yes	59	15	25.4	6	10.2
	No	5973	2167	36.3	712	11.9
Immunotherapy^a^	Yes	27	18	66.7	11	40.7
	No	6003	2162	36.3	706	11.8
Insurance^a,c^	Private only	2813	720	25.6	254	9.0
	Medicare only, or Medicare + private	1816	824	45.4	250	13.8
	Medicaid/military/other public	727	262	36.0	86	11.8
	None/self pay	68	11	16.2	8	11.8

Data are presented as number of patients and % of total unless otherwise indicated. Percentages of death and death from lung cancer are provided per total population within the subgroup.

^a^Counts do not add up to 6038 due to missing data. ^b^Radiation treatment data was collected through year 2017 only. ^c^Payer/insurance carrier was not mandatory to report in CCR prior to 1996, so insurance data is presented for the subset of *n* = 5527 cases diagnosed from 1996 onward.

IQR, interquartile range; NETs, neuroendocrine tumor; NH, non-Hispanic; SES, socioeconomic status.

### Characteristics of study population by stage at diagnosis

We compared demographic, clinical, and disease characteristics of the study population by stage at diagnosis (Table 2). Compared to cases diagnosed with localized or regional disease, cases with distant metastases at diagnosis had larger tumors and were older, more likely to be diagnosed in the most recent decade, more likely to have atypical carcinoid histology, more likely to be non-Hispanic Black, more likely to reside in neighborhoods in the lowest nSES quintile, more likely to have comorbidities, and less likely to have private health insurance (all *P* < 0.001 for differences by stage). The breakdown of stage at diagnosis by histology was similar for the subset of cases diagnosed after 1997 (data not shown). Stage at diagnosis did not differ by sex, county of residence, or marital status. Characteristics of the study population by histology are shown in (Supplementary Table 1, see section on supplementary materials given at the end of this article).

**Table 2:** Demographic and clinical characteristics of lung NET population by stage at diagnosis.

Variable	Level	Local/regional disease (*n* = 5084)	Metastatic disease (*n* = 739)	*P* value^a^
Age at diagnosis	Median (IQR)	63 (52, 72)	68 (58, 77)	**<0.001**
Diagnosis decade	1992–2000	1114 (90.9%)	112 (9.1%)	**<0.001**
	2001–2009	1516 (87.7%)	212 (12.3%)	
	2010–2019	2454 (85.5%)	415 (14.5%)	
Sex^b^	Male	1533 (87.6%)	218 (12.4%)	0.71
	Female	3550 (87.2%)	521 (12.8%)	
Race/ethnicity^b^	NH White	3773 (88.2%)	504 (11.8%)	**<0.001**
	Hispanic	804 (86.7%)	123 (13.3%)	
	NH Black	241 (79.8%)	61 (20.2%)	
	Asian/Pacific Islander	222 (83.8%)	43 (16.2%)	
	American Indian	24 (82.8%)	5 (17.2%)	
County	Urban	3664 (87.8%)	508 (12.2%)	0.16
	Suburban	1284 (85.9%)	211 (14.1%)	
	Rural	136 (87.2%)	20 (12.8%)	
Marital status^b^	Unmarried	2058 (87.3%)	299 (12.7%)	0.85
	Married	2900 (87.5%)	415 (12.5%)	
Neighborhood SES at diagnosis^b^	Quintile 1 (lowest nSES)	536 (83.4%)	107 (16.6%)	**<0.001**
	Quintile 2	844 (85.3%)	145 (14.7%)	
	Quintile 3	1117 (86.2%)	178 (13.8%)	
	Quintile 4	1239 (88.3%)	164 (11.7%)	
	Quintile 5 (highest nSES)	1347 (90.3%)	739 (12.7%)	
Histology	Typical carcinoid	4740 (88.3%)	627 (11.7%)	**<0.001**
	Atypical carcinoid	344 (75.4%)	112 (25.6%)	
Charlson comorbidities index^b^	None	2317 (90.2%)	252 (9.8%)	**<0.001**
	1–2	1753 (86.6%)	272 (13.4%)	
	≥3	379 (82.8%)	79 (17.2%)	
Primary tumor size (cm)	Median (IQR)	2.0 (1.3, 2.8)	2.5 (1.5, 4.3)	**<0.001**
Insurance^b,c^	Private only	2463 (89.3%)	294 (10.7%)	**<0.001**
	Medicare only, or Medicare + private	1498 (85.4%)	255 (14.6%)	
	Medicade/military/other public	578 (81.8%)	129 (18.2%)	
	None/self pay	52 (81.2%)	12 (18.8%)	

Demographic and clinical characteristics for the 5823 patients with stage at diagnosis information available. Data are presented as number of patients (%) unless otherwise indicated.

^a^*P*-value for difference between local/regional disease and distant metastatic disease at diagnosis obtained from chi-square test for categorical variables or Wilcoxon rank sum test for continuous variables. Bold indicates statistical significance. ^b^Counts do not add up to 5823 due to missing data. ^c^Payer/insurance carrier reporting was not mandatory in the California Cancer Registry prior to 1996, so insurance data is presented for the *n* = 5348 cases diagnosed after 1995.

### Univariate OS

**Figure 1:**
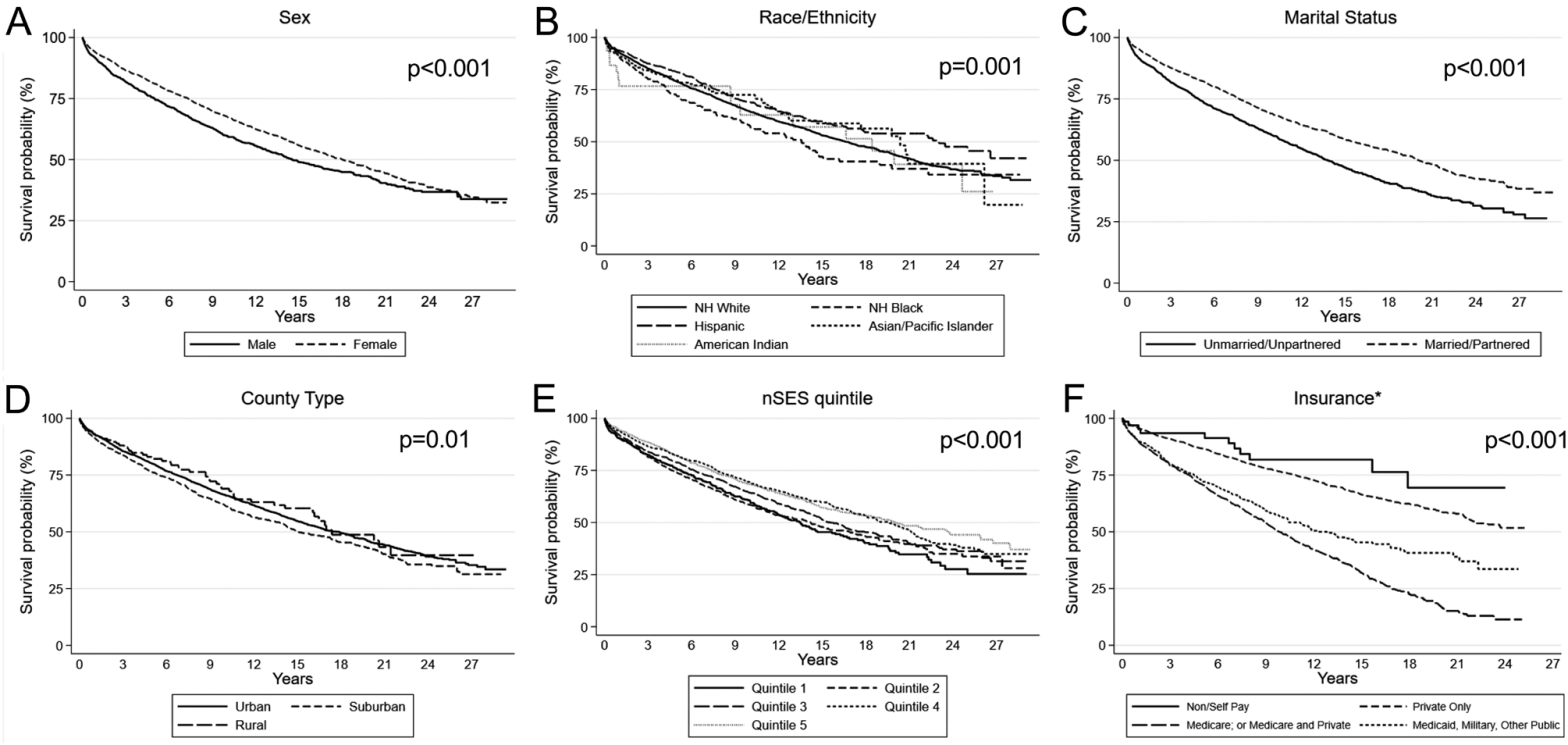
Kaplan–Meier overall survival curves by sociodemographic variables. NH, non-Hispanic, nSES, neighborhood socioeconomic status. *Insurance payer was not required in CCR prior to 1996, so insurance data are presented for the *n* = 5527 cases diagnosed from 1996 onward.

**Figure 2:**
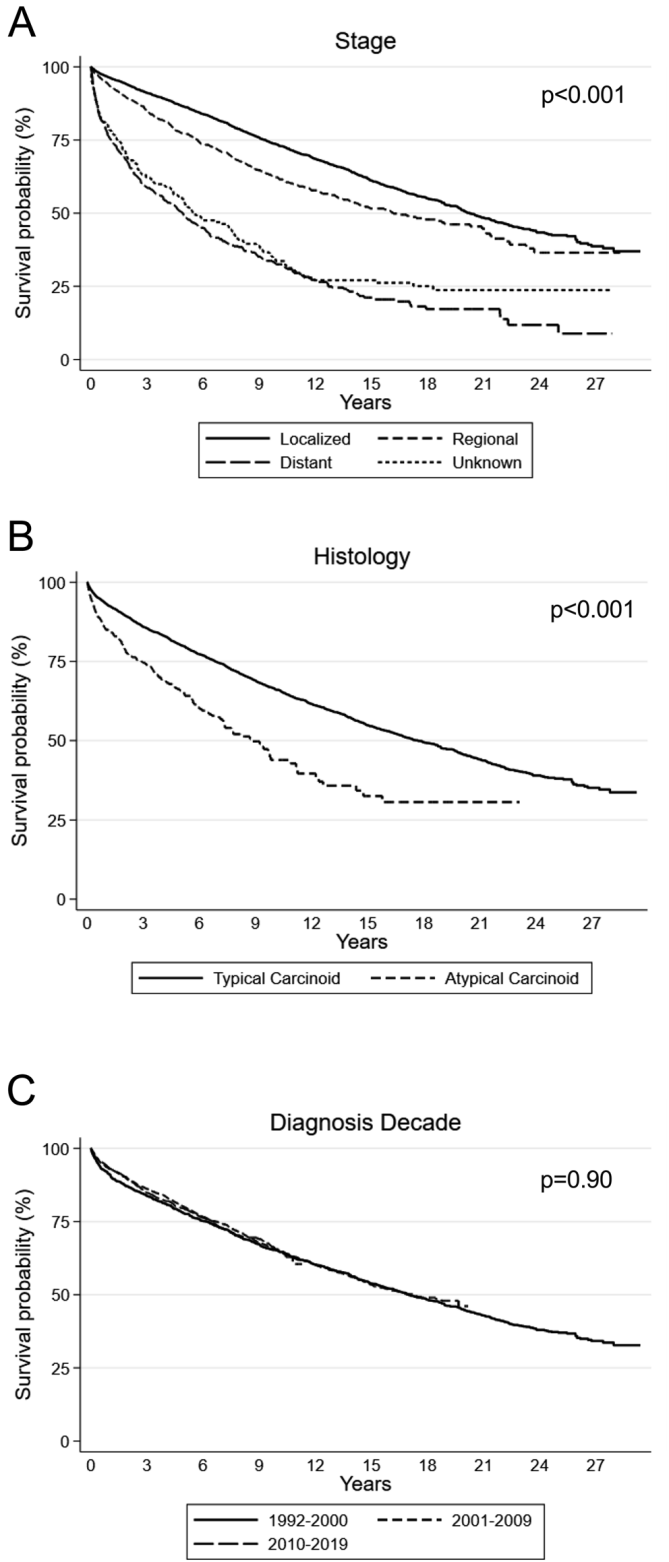
Kaplan–Meier overall survival curves by disease characteristics.

Kaplan–Meier survival analyses showed statistically significant differences in survival by sociodemographic factors and social determinants of health (Fig. 1). OS was worse for men vs women (Fig. 1A) for non-Hispanic Black cases compared with non-Hispanic White (*P* = 0.014) or Hispanic (*P* < 0.001) cases (Fig. 1B), for unmarried cases (Fig. 1C), for cases living in suburban counties (Fig. 1D), and for cases living in the lowest nSES quintile (Fig. 1E). For cases diagnosed after 1995, compared to people with private health insurance, people with Medicare had worse survival (*P* < 0.001), as did those with Medicaid, military, or other public insurance (*P* < 0.001; Fig. 1F). We also confirmed associations of stage (Fig. 2A) and histology (Fig. 2B) with OS. We found similar differences in survival by histology for the subset of cases diagnosed after 1997 (data not shown). In contrast, OS did not differ by diagnosis decade (Fig. 2C).

### Multivariable OS

Next, we examined sequential, age-stratified multivariable models of OS to understand the direct impact of sociodemographic factors on mortality independent of each other and disease- or treatment-related variables (Table 3). Women had better OS compared with men across all models. Non-Hispanic Black cases had worse OS compared with non-Hispanic White cases in an age-stratified univariate Cox model (HR 1.52, 95% CI 1.27–1.82, *P* < 0.001). However, the OS difference for non-Hispanic Black cases was no longer significant in our multivariable models, largely mediated by stage at diagnosis (Supplementary Table 2), with non-Hispanic Black cases more likely to have metastatic disease compared with other racial and ethnic groups (Table 2). Asian-American/Pacific Islander cases had better OS compared with non-Hispanic White cases in our fully adjusted OS model only. Cases who were partnered at diagnosis had better OS than unmarried cases across all models. Residence in a high vs low nSES quintile at diagnosis was also associated with lower all-cause mortality across all models. In contrast, county of residence was not generally associated with OS after adjustment for covariates.

**Table 3:** Associations between sociodemographic, clinicopathologic, and treatment factors with all-cause mortality in age-stratified models among *n* = 6038 lung NETs, 1992–2019.

Variable	Level	Model 1		Model 2		Model 3		Model 4^a^	
		HR (95% CI)	*P-*value	HR (95% CI)	*P-*value	HR (95% CI)	*P-*value	HR (95% CI)	*P-*value
Sex	Male	1	–	1	–	1	–	1	–
	Female	0.59 (0.54, 0.65)	**<0.001**	0.60 (0.54, 0.65)	**<0.001**	0.62 (0.57, 0.68)	**<0.001**	0.61 (0.55, 0.67)	**<0.001**
Race/ethnicity	NH White	1	–	1	–	1	–	1	–
	Hispanic	0.94 (0.82, 1.07)	0.34	0.94 (0.82, 1.08)	0.38	0.90 (0.79, 1.04)	0.14	0.89 (0.77, 1.03)	0.12
	NH Black	1.18 (0.98, 1.42)	0.073	1.16 (0.96, 1.39)	0.12	0.99 (0.83, 1.20)	0.95	0.96 (0.79, 1.17)	0.71
	Asian/Pacific Islander	0.90 (0.72, 1.13)	0.28	0.88 (0.70, 1.11)	0.29	0.77 (0.61, 0.97)	**0.025**	0.69 (0.53, 0.88)	**0.003**
	American Indian	1.33 (0.79, 2.22)	0.28	1.32 (0.78, 2.21)	0.29	1.32 (0.79, 2.21)	0.29	1.98 (1.14, 3.44)	**0.013**
County	Urban	1	–	1	–	1	–	1	–
	Suburban	1.12 (1.01, 1.23)	**0.024**	1.10 (1.0, 1.21)	0.051	1.07 (0.97, 1.18)	0.20	1.04 (0.94, 1.16)	0.45
	Rural	0.83 (0.63, 1.10)	0.19	0.82 (0.62, 1.08)	0.16	0.82 (0.62, 1.08)	0.16	0.81 (0.60, 1.10)	0.17
Marital status	Unmarried	1	–	1	–	1	–	1	–
	Married	0.74 (0.68, 0.81)	**<0.001**	0.73 (0.67, 0.80)	**<0.001**	0.76 (0.70, 0.84)	**<0.001**	0.76 (0.69, 0.84)	**<0.001**
nSES	Quintile 1 (lowest)	1	–	1	–	1	–	1	–
	Quintile 2	1.00 (0.86, 1.18)	0.94	1.01 (0.86, 1.18)	0.93	0.99 (0.85, 1.16)	0.92	1.08 (0.91, 1.28)	0.40
	Quintile 3	0.92 (0.79, 1.07)	0.28	0.92 (0.79, 1.08)	0.31	0.93 (0.80, 1.08)	0.33	0.97 (0.82, 1.15)	0.72
	Quintile 4	0.77 (0.66, 0.90)	**0.001**	0.78 (0.66, 0.91)	**0.001**	0.77 (0.66, 0.90)	**0.001**	0.83 (0.70, 0.99)	**0.039**
	Quintile 5 (highest nSES)	0.72 (0.62, 0.84)	**<0.001**	0.72 (0.62, 0.84)	**<0.001**	0.73 (0.62, 0.85)	**<0.001**	0.80 (0.67, 0.94)	**0.008**
Stage	Local	1	–	1	–	1	–	1	–
	Regional	1.50 (1.34, 1.69)	**<0.001**	1.47 (1.31, 1.65)	**<0.001**	1.33 (1.18, 1.49)	**<0.001**	1.27 (1.12, 1.45)	**<0.001**
	Distant	3.46 (3.09, 3.88)	**<0.001**	3.43 (3.06, 3.84)	**<0.001**	2.15 (1.88, 2.44)	**<0.001**	2.08 (1.82, 2.39)	**<0.001**
	Unknown	2.36 (1.96, 2.84)	**<0.001**	2.33 (1.93, 2.80)	**<0.001**	1.42 (1.17, 1.73)	**0.001**	1.32 (1.05, 1.65)	**0.016**
Diagnosis decade	1992-2000	1	–	1	–	1	–	1	–
	2001-2009	0.77 (0.69, 0.85)	**<0.001**	0.74 (0.66, 0.82)	**<0.001**	0.71 (0.64, 0.80)	**<0.001**	0.75 (0.66, 0.85)	**<0.001**
	2010-2019	0.70 (0.62, 0.79)	**<0.001**	0.64 (0.57, 0.73)	**<0.001**	0.63 (0.55, 0.72)	**<0.001**	0.66 (0.57, 0.77)	**<0.001**
Histology	Typical carcinoid			1	–	1	–	1	–
	Atypical carcinoid			1.86 (1.58, 2.18)	**<0.001**	1.78 (1.51, 2.09)	**<0.001**	1.79 (1.52, 2.11)	**<0.001**
Treatment	Surgery (no)					1	–	1	–
	Surgery (yes)					0.48 (0.43, 0.54)	**<0.001**	0.49 (0.43, 0.55)	**<0.001**
	Radiation (no)					1	**–**	1	**–**
	Radiation (yes)					1.51 (1.28, 1.78)	**<0.001**	1.49 (1.24, 1.78)	**<0.001**
	Chemotherapy (no)					1	–	1	–
	Chemotherapy (yes)					1.90 (1.60, 2.25)	**<0.001**	1.94 (1.62, 2.31)	**<0.001**
	Chemotherapy (unknown)					1.85 (1.19, 2.89)	**0.006**	2.11 (1.30, 3.41)	**0.002**
	Hormone treatment (no)					1	–	1	–
	Hormone treatment (yes)					0.71 (0.42, 1.19)	0.19	0.72 (0.43, 1.20)	0.20
	Immune treatment (no)					1	–	1	–
	Immune treatment (yes)					1.81 (1.12, 2.92)	**0.015**	1.69 (1.00, 2.85)	**0.049**
Insurance	Private only							1	–
	Medicare only or Medicare + private							1.24 (1.10, 1.40)	**<0.001**
	Medicaid/military/other public							1.45 (1.24, 1.68)	**<0.001**
	None/self pay							0.68 (0.37, 1.25)	0.21

Multivariable Cox regression models of overall survival. All models were stratified by age. Model 1 was adjusted for sociodemographic and basic disease characteristics, including sex, race/ethnicity, county, marital status, nSES, stage, and decade of diagnosis. Model 2 was adjusted for the variables in model 1, plus histology. Model 3 was adjusted for the variables in model 2, plus treatment variables. Model 4 was adjusted for the variables in model 3, plus insurance payer. Bold indicates statistical significance, *P* < 0.05.

^a^Model 4 includes only the *n* = 5527 cases diagnosed after 1995, when collection of insurance payer information was mandated in the California Cancer Registry.

CI, confidence interval; NH, non-Hispanic; HR, hazard ratio; nSES, neighborhood socioeconomic status.

Beyond sociodemographic factors, we found significant differences in OS by disease- and treatment-related factors. Across all models, we confirmed worse OS for cases with regional or distant stage at diagnosis compared with localized disease. Atypical carcinoid histology was associated with worse OS compared with typical carcinoid histology. After adjustment for sociodemographic and disease characteristics, we found improved OS for cases diagnosed in the more recent two decades compared with the earliest decade, and this association persisted after further adjustment for treatment variables. In terms of treatments, surgery was strongly associated with better OS. In contrast, radiation and chemotherapy were associated with higher mortality. Finally, immune therapies were also associated with worse OS, although the number of patients who received these treatments was small. In additional sensitivity analyses (data not shown), the significance and scope of our multivariable findings in Table 3 did not change if we restricted our fully adjusted model 3 to only those cases diagnosed after 1997. We also found similar associations between sociodemographic factors, disease characteristics, and treatments with OS in an additional fully adjusted model that included the Charlson comorbidity index as a covariate (Supplementary Table 3).

To examine associations of insurance with mortality, we ran an additional fully adjusted, age-stratified model (model 4) that included health insurance payer as a covariate for the subset of cases diagnosed after 1995. Compared with private insurance, we found worse OS for cases with Medicare (HR 1.24, 95% CI 1.10–1.40, *P* < 0.001) or Medicaid, military, or other public insurance (HR 1.45, 95% CI 1.24–1.68, *P* < 0.001). All other sociodemographic and clinicopathologic variables that were previously associated with OS remained significant in this model even after further adjustment for insurance.

We found heterogeneity in the effect of county of residence on OS by nSES (heterogeneity *P* = 0.01 in fully adjusted model 3). For the low nSES stratum (defined as statewide nSES quintiles 1, 2, or 3), compared with cases residing in urban counties, cases residing in suburban counties had worse survival (HR 1.19, 95% CI 1.05–1.35, *P* = 0.007). In contrast, for the high nSES stratum (defined as nSES quintile 4 or 5), there were no differences in OS by rural/urban status.

### LCSS

Finally, we ran multivariable Fine-Gray competing risks regression models to examine associations between sociodemographic, clinicopathologic, and treatment factors with LCSS (Table 4). Age, sex, marital status, stage, diagnosis decade, and histology were all significantly associated with LCSS. American Indian cases had worse LCSS across all models. Unlike for OS, nSES and insurance were not associated with LCSS. Treatment with surgery, radiation, and chemotherapy were also associated with LCSS in the same direction as for OS. Overall associations were similar in an additional fully adjusted LCSS model that included the Charlson comorbidity index as a covariate (Supplementary Table 4).

**Table 4:** Associations between sociodemographic, clinicopathologic, and treatment factors with lung cancer-specific mortality in completing-risks regression models among *n* = 6038 lung NETs, 1992–2019.

Variable	Level	Model 1		Model 2		Model 3		Model 4^a^	
		SHR (95% CI)	*P* value	SHR (95% CI)	*P* value	SHR (95% CI)	*P* value	SHR (95% CI)	*P* value
Age at diagnosis	<45	1	–	1	–	1	–	1	–
	45–54	1.93 (1.29, 2.90)	**0.002**	1.93 (1.28, 2.89)	**0.002**	1.86 (1.24, 2.77)	**0.002**	1.90 (1.19, 3.03)	**0.007**
	55–65	3.06 (2.12, 4.43)	**<0.001**	3.14 (2.17, 4.53)	**<0.001**	2.71 (1.87, 3.93)	**<0.001**	2.88 (1.87, 4.43)	**<0.001**
	65–74	3.82 (2.65, 5.49)	**<0.001**	3.87 (2.69, 5.57)	**<0.001**	3.49 (2.42, 5.02)	**<0.001**	3.84 (2.48, 5.96)	**<0.001**
	≥75	4.67 (3.43, 7.18)	**<0.001**	5.11 (3.53, 7.39)	**<0.001**	4.20 (2.88, 6.13)	**<0.001**	4.59 (2.93, 7.21)	**<0.001**
Sex	Male	1	–	1	–	1	–	1	–
	Female	0.58 (0.49, 0.68)	**<0.001**	0.60 (0.51, 0.70)	**<0.001**	0.65 (0.55, 0.77)	**<0.001**	0.65 (0.54, 0.77)	**<0.001**
Race/ethnicity	NH White	1	–	1	–	1	–	1	–
	Hispanic	0.89 (0.69, 1.14)	0.34	0.90 (0.71, 1.16)	0.42	0.86 (0.67, 1.11)	0.25	0.81 (0.62, 1.06)	0.13
	NH Black	1.37 (1.02-1.83)	**0.036**	1.33 (1.00, 1.76)	**0.049**	1.06 (0.78, 1.43)	0.73	1.09 (0.79, 1.48)	0.61
	Asian/Pacific Islander	1.10 (0.74, 1.63)	0.65	1.07 (0.72, 1.58)	0.74	0.85 (0.55, 1.30)	0.45	0.84 (0.53, 1.32)	0.45
	American Indian	2.27 (1.02, 5.06)	**0.046**	2.38 (1.11, 5.09)	**0.026**	2.34 (1.15, 4.80)	**0.020**	3.17 (1.56, 6.43)	**0.001**
County	Urban	1	–	1	–	1	–	1	–
	Suburban	1.18 (0.99, 1.40)	0.059	1.16 (0.98, 1.38)	0.085	1.10 (0.92, 1.31)	0.28	1.12 (0.92, 1.35)	0.26
	Rural	0.79 (0.47, 1.33)	0.38	0.75 (0.46, 1.25)	0.27	0.77 (0.47, 1.27)	0.31	0.64 (0.36, 1.15)	0.14
Marital status	Unmarried	1	–	1	–	1	–	1	–
	Married	0.75 (0.64, 0.88)	**0.001**	0.73 (0.62, 0.86)	**<0.001**	0.76 (0.64, 0.89)	**0.001**	0.81 (0.68, 0.96)	**0.017**
nSES	Quintile 1 (lowest nSES)	1	–	1	–	1	–	1	–
	Quintile 2	1.32 (0.98, 1.76)	0.067	1.32 (0.99, 1.77)	0.061	1.28 (0.95, 1.72)	0.10	1.28 (0.93, 1.76)	0.13
	Quintile 3	1.25 (0.94, 1.67)	0.12	1.27 (0.96, 1.68)	0.097	1.26 (0.94, 1.67)	0.12	1.23 (0.90, 1.66)	0.19
	Quintile 4	0.94 (0.70, 1.27)	0.68	0.93 (0.69, 1.25)	0.64	0.88 (0.65, 1.19)	0.40	0.89 (0.64, 1.23)	0.49
	Quintile 5 (highest nSES)	0.94 (0.70, 1.27)	0.69	0.94 (0.70, 1.27)	0.69	0.91 (0.67, 1.24)	0.55	0.89 (0.64, 1.25)	0.51
Stage	Local	1	–	1	–	1	–	1	–
	Regional	2.75 (2.26, 3.35)	**<0.001**	2.61 (2.14, 3.18)	**<0.001**	2.18 (1.77, 2.68)	**<0.001**	2.04 (1.63, 2.56)	**<0.001**
	Distant	6.86 (5.68, 8.29)	**<0.001**	6.56 (5.42, 7.93)	**<0.001**	3.43 (2.76, 4.28)	**<0.001**	3.28 (2.61, 4.14)	**<0.001**
	Unknown	3.09 (2.21, 4.33)	**<0.001**	3.03 (2.17, 4.23)	**<0.001**	1.73 (1.22, 2.46)	**0.002**	1.51 (1.01, 2.26)	**0.044**
Diagnosis decade	1992-2000	1	–	1	–	1	–	1	–
	2001-2009	0.65 (0.54, 0.79)	**<0.001**	0.59 (0.48, 0.71)	**<0.001**	0.61 (0.51, 0.75)	**<0.001**	0.69 (0.55, 0.86)	**0.001**
	2010-2019	0.47 (0.39, 0.58)	**<0.001**	0.37 (0.30, 0.46)	**<0.001**	0.40 (0.32, 0.49)	**<0.001**	0.44 (0.34, 0.56)	**<0.001**
Histology	Typical carcinoid			1	–	1	–	1	–
	Atypical carcinoid			3.08 (2.44, 3.89)	**<0.001**	2.71 (2.11, 3.47)	**<0.001**	2.77 (2.15, 3.56)	**<0.001**
Treatment	Surgery (no)					1	–	1	–
	Surgery (yes)					0.46 (0.38, 0.56)	**<0.001**	0.45 (0.37, 0.55)	**<0.001**
	Radiation (no)					1	–	1	–
	Radiation (yes)					1.79 (1.40, 2.30)	**<0.001**	1.94 (1.49, 2.53)	**<0.001**
	Chemotherapy (no)					1	–	1	–
	Chemotherapy (yes)					2.12 (1.63, 2.75)	**<0.001**	2.10 (1.60, 2.76)	**<0.001**
	Chemotherapy (unknown)					2.84 (1.54, 5.24)	**0.001**	3.91 (2.26, 6.78)	**<0.001**
	Hormone treatment (no)					1	–	1	–
	Hormone treatment (yes)					0.56 (0.26, 1.23)	0.15	0.60 (0.27, 1.32)	0.20
	Immune treatment (no)					1	–	1	–
	Immune treatment (yes)					1.49 (0.82, 2.72)	0.20	1.26 (0.61, 2.57)	0.53
Insurance	Private only							1	–
	Medicare only or Medicare + Private							1.05 (0.85, 1.30)	0.67
	Medicaid/military/other public							1.07 (0.82, 1.41)	0.52
	None/self pay							1.82 (0.90, 3.70)	0.098

Competing risks regression models of lung cancer specific survival with subdistribution hazard ratios obtained from the fine-gray model. Model 1 was adjusted for sociodemographic and basic disease characteristics, including age, sex, race/ethnicity, county, marital status, nSES, stage, and decade of diagnosis. Model 2 was adjusted for the variables in model 1, plus histology. Model 3 was adjusted for the variables in model 2, plus treatment variables. Model 4 was adjusted for the variables in model 3, plus insurance payer. Bold indicates statistical significance, *P* < 0.05.

^a^Model 4 includes only the *n* = 5527 cases diagnosed after 1995, when collection of insurance payer information was mandated in the California Cancer Registry.

CI, confidence interval; NH, non-Hispanic; nSES, neighborhood socioeconomic status; SHR, subhazard ratio.

## Discussion

Lung NET epidemiology has been studied less than other primary sites. Nearly all are sporadic, although rarely they are associated with familial genetic syndromes (Oliveira *et al.* 2001). The contribution of chronic inflammation, underlying pulmonary conditions like asthma or chronic obstructive pulmonary disease, or environmental factors like smoking are poorly understood. Some patients develop diffuse idiopathic pulmonary neuroendocrine cell hyperplasia as a precursor to lung NETs but not a majority (Samhouri *et al.* 2023). Both the underlying mechanisms driving NET pathogenesis and clinical outcomes may vary by case.

Here, we report a comprehensive summary of patients diagnosed with lung NETs in California’s population-based cancer registry. More cases were diagnosed in the most recent decade of our study compared with the earliest decade, including more with metastatic disease. Underlying contributors to this finding may include better detection of advanced disease by imaging, improved recognition of NET histologies by pathologists, and potentially rising incidence.

Women were a majority of the lung NET population, consistent with prior studies (Steuer *et al.* 2015, Shah *et al.* 2021). The reasons for this sex difference are unclear. Women might be diagnosed more frequently than men as a consequence of higher healthcare-seeking behavior and more contact with the healthcare system (Bertakis *et al.* 2000). However, lung cancer screening by low-dose computed tomography is equally infrequent in women and men (Jemal & Fedewa 2017), and recent screening recommendations would not explain our findings across all decades. Furthermore, we found no difference by sex in stage at diagnosis, suggesting that increased early-stage detection does not explain why lung NETs occur predominantly in women. A biologic mechanism for the sex difference is possible, perhaps through a role for hormones like estrogen in regulating gene expression and promoting neuroendocrine cell proliferation. There may be unrecognized differences in behavior or risk factors that predispose women to lung NETs. Further study is merited to clarify the mechanism.

We observed that lung NET diagnoses are associated with race and ethnicity along with several social determinants of health. Californians self-identifying as non-Hispanic White were a higher proportion of our lung NET cohort compared to statewide demographics (Humes *et al.* 2011), while other racial and ethnic groups were under-represented in the lung NET population, particularly Hispanic and Asian/Pacific Islander. Non-Hispanic White cases were less likely to have metastatic disease at diagnosis compared with other racial/ethnic groups. People living in the highest statewide nSES quintile were also over-represented in the lung NET cohort, and they were less likely to have metastatic disease at diagnosis compared with the lowest quintile. Finally, people with private health insurance were less likely to have metastatic disease compared to those with public or no insurance.

The impact of sociodemographic factors on NET diagnoses and stage at diagnosis could be an artifact of increased detection of early-stage cancer in privileged groups and a corresponding underdiagnosis of lung NETs (particularly early-stage) in vulnerable populations, including people of color, people living in economically disadvantaged communities, and people with public or no health insurance. NETs can be challenging to diagnose, even for experienced pathologists. Lung NETs are occasionally misclassified as other malignancies (Kasajima *et al.* 2022), and this misclassification may be more common at medical centers that serve vulnerable populations. Alternatively, sociodemographic differences could be explained by underlying risk factors and/or health-related behaviors associated with both social determinants of health and the pathogenesis of lung NETs. Importantly, we do not believe a genetic explanation for the racial and ethnic makeup of the lung NET cohort is likely, given that the vast majority of cases are sporadic, and considering the lack of consistent correlation between self-identified race and ethnicity, genetic ancestry, and genomic variants (Bryc *et al.* 2015).

We also report an in-depth analysis of predictors of OS and LCSS for lung NETs in California. Prior studies demonstrated that age, histology, and stage are associated with survival, with worse OS for older patients, atypical vs typical carcinoid histology, and metastatic compared with localized disease (García-Yuste *et al.* 2007, Filosso *et al.* 2015). We confirmed these associations in our multivariable analyses. In addition, we identified several sociodemographic factors also associated with survival. As reported in another SEER study (Shah *et al.* 2021), we found that sex, marital status, and health insurance were associated with OS. Women had better OS than men, even after adjusting for other prognostic factors including sociodemographic factors, disease characteristics, and first course of treatment. There may be differences in biology and other underlying risk factors by sex; for example, we found that primary tumor size differed between women (median 1.8 cm, IQR 1.2, 2.7) and men (median 2.3 cm, IQR 1.5, 3.5; *P* < 0.001). Non-Hispanic Black Californians had worse OS than non-Hispanic White Californians in a univariate model, consistent with one prior study of atypical carcinoid tumors (Steuer *et al.* 2015). However, this survival difference was eliminated after adjustment for other prognostic factors and appeared to be mediated by disease characteristics (in particular, stage at diagnosis) along with other social determinants of health.

We also report a new finding that nSES was strongly predictive of OS in lung NETs, with lower all-cause mortality for cases living in higher nSES quintiles. This association persisted despite adjustment for other sociodemographic, disease, and treatment-related variables, suggesting that the environment in which one lives has implications for longevity after a lung NET diagnosis.

As reported in a prior study, we found a clear survival benefit for married/partnered individuals (Shah *et al.* 2021). Interestingly, stage at diagnosis did not differ by marital status, so the survival benefit is not explained by earlier detection alone. Health insurance was also independently associated with OS. One prior study reported worse survival for people with insurance compared to the uninsured but did not provide granular information about insurance type (Shah *et al.* 2021). In our analyses, we found worse OS for people with Medicare or public health insurance compared to those with private insurance only, even after stratifying by age and adjustment for disease and treatment variables.

As many lung NET patients experience prolonged survival, the longitudinal risk of noncancer death is important to consider when assessing outcomes. Our analysis of LCSS revealed both similarities and differences compared with OS associations. Non-Hispanic Black cases had worse LCSS in our partially adjusted model including stage, but this attenuated after adjustment for histology, likely reflecting how non-Hispanic Black cases were more likely to have atypical carcinoid histology (Supplementary Table 1). We also report a novel finding that American Indian cases (a group not previously well studied for NET outcomes) had worse LCSS compared with non-Hispanic White cases. This finding should be replicated in additional studies, as the number of cases in our dataset was small. As with our OS analysis, we found that sex and marital status were strongly associated with LCSS across all models. However, neither nSES nor insurance was associated with LCSS in any multivariate models; these variables may be important predictors of general health outcomes rather than lung NET-specific mortality.

It comes as no surprise that social determinants of health are associated with survival. Given that lung NETs often progress over years, there is ample time for social factors to compound and impact outcomes. Marital status has been associated with survival across a range of malignancies (Aizer *et al.* 2013) and can be a proxy for socioeconomic resources and other health-related factors including diet, physical activity, preventative care, and more aggressive treatment. Interestingly, the association between marital status and survival was not attenuated by controlling for nSES, treatments, or health insurance. Similarly, SES is a fundamental cause of health. Higher SES individuals can flexibly deploy resources to avoid health risks and mitigate the effects of poor health. One limitation of our study is the absence of an individual-level measure of SES. However, nSES may influence health through distinct mechanisms than individual SES; for example, through access to medical care, quality of education, environmental exposures, crime, crowding, recreational opportunities, and municipal services.

Based on a prior population-based study in Ontario, Canada (Hallet *et al.* 2015), and a SEER publication in the United States (Shah *et al.* 2021), we hypothesized that rural residents in California would have worse survival. Instead, univariate models suggested that suburban, not rural, residents have the worst OS, and there were no associations between county of residence and survival in either multivariable OS or LCSS models. We also found heterogeneity by nSES in the associations between rural/urban status and mortality. Californians of low nSES had worse OS in suburban counties compared with urban counties. In contrast, for Californians of high nSES, there were no survival differences by county. The reasons for worse survival for low nSES residents of suburbs may reflect differences in access to healthcare resources and/or differences in chronic medical conditions, health-related behaviors, or other aspects of the built environment. Compared to suburban residents, rural residents may be referred more often to NET centers of expertise, which have been shown to improve NET outcomes (Baeg *et al.* 2021). In contrast, socioeconomically advantaged people may find their way to NET centers of expertise regardless of where they live.

Beyond sociodemographic and clinicopathologic factors, we also confirmed strong associations between lung NET treatments and survival. Surgery was associated with improved OS and LCSS. This finding is consistent with existing literature; surgical resection is the primary treatment for localized lung NETs and is usually done with curative intent (Raz *et al.* 2015, Steuer *et al.* 2015). Chemotherapy and radiation were associated with worse OS and LCSS, while immune therapy was associated with worse OS but not LCSS. These treatment associations may reflect residual confounding due to disease aggressiveness and patient comorbidities. In particular, chemotherapy is generally reserved for lung NETs with the most aggressive biology, so we interpret the association with higher mortality as a reflection of disease severity rather than the role of chemotherapy itself. Conversely, patients who undergo surgery are likely healthier overall with a better prognosis. Finally, we found improved OS and LCSS in the more recent two decades of our study compared with the earliest decade, even though cases diagnosed in the most recent decade were more likely to have metastatic disease. This association was independent of treatment variables. However, because the CCR only captures treatment during the first year after diagnosis and many lung NET patients have extended survival, it is still possible that treatment advances may explain recent improvements in survival.

This study has several strengths. As a population-based registry that captures new cancer diagnoses across California, the CCR is less subject to selection bias compared with cases series from single institutions. California is a large and diverse state that is well-suited to address questions of disparities. Our study also has limitations. Given that we only included cases in California, our conclusions may not be generalizable to the broader United States or global population with lung NETs. Because NETs are challenging to diagnose and classify, there is likely some selection bias for who is diagnosed. The classification of tumor grade for NETs has also changed over time, so there is likely some degree of histologic misclassification in the dataset. Because we relied on topography to determine lung NET-specific cause of death, it is possible we overestimated hazards for non-NET cancers arising in the lung. Furthermore, as an observational study, our results are subject to residual confounding. We attempted to control for many potential confounders, but we were limited to information collected in the CCR. Our analysis could not account for treatments throughout the duration of illness, or the impact of other comorbidities and lifestyle factors such as smoking, disease-related factors like Ki-67/mitotic count, or hormone overproduction/functional status on outcomes. Lead time bias may also impact the observed outcome differences of various groups. Type I error is also possible due to multiple comparisons. However, while chance may have theoretically played a role, the consistency of our findings across multiple models is reassuring, and our results have both biologic plausibility and supportive data in other cancer types.

In conclusion, we report novel findings that sociodemographic factors, along with clinicopathologic and treatment factors, are associated with OS and, to a lesser extent, LCSS in lung NETs. We believe these results will influence future research into the pathogenesis of lung NETs and help identify opportunities for interventions to reduce disparities and improve survival for these increasingly common malignancies.

## Supplementary Materials

Supplementary Tables

## Declaration of interest

CKM has served as an advisor to Jazz Pharmaceuticals and received research funding to her institution as local clinical trial principal investigator from RayzeBio. MAG has served as an advisor to AnHeart, AstraZeneca, BMS, Cardinal Health, Genentech/Roche, Genzyme, Gilead, Guardant, iTeos, Sanofi, Summit, and Surface and received research funding to his institution as local clinical trial principal investigator from Amgen, Celgene, JNJ, Merck, Novartis, OncoMed, and Trizell. All other authors declare no conflicts of interest.

## Funding

This work was supported by a Mentored Award from the Neuroendocrine Tumor Research Foundationhttp://dx.doi.org/10.13039/100009863 (grant number 654559).

## References

[bib1] Aizer AA Chen MH McCarthy EP Mendu ML Koo S Wilhite TJ Graham PL Choueiri TK Hoffman KE Martin NE, *et al.* 2013 Marital status and survival in patients with cancer. Journal of Clinical Oncology 31 3869–3876. (10.1200/JCO.2013.49.6489) 24062405 PMC4878087

[bib2] Baeg K Harris C Naparst MS Ahn E Thapi S Martin J Rustgi S Mhango G Wisnivesky J & Kim MK 2021 Effect of treatment center volume on outcomes in gastroenteropancreatic neuroendocrine tumor patients. BMC Cancer 21 146. (10.1186/s12885-021-07868-8) 33563241 PMC7871611

[bib3] Bertakis KD Azari R Helms LJ Callahan EJ & Robbins JA 2000 Gender differences in the utilization of health care services. Journal of Family Practice 49 147–152. 10718692

[bib4] Bryc K Durand EY Macpherson JM Reich D & Mountain JL 2015 The genetic ancestry of African Americans, Latinos, and European Americans across the United States. American Journal of Human Genetics 96 37–53. (10.1016/j.ajhg.2014.11.010) 25529636 PMC4289685

[bib5] Fernandez-Cuesta L Peifer M Lu X Sun R Ozretić L Seidel D Zander T Leenders F George J Müller C, *et al.* 2014 Frequent mutations in chromatin-remodelling genes in pulmonary carcinoids. Nature Communications 5 3518. (10.1038/ncomms4518) PMC4132974 24670920

[bib6] Filosso PL Guerrera F Evangelista A Welter S Thomas P Casado PM Rendina EA Venuta F Ampollini L Brunelli A, *et al.* 2015 Prognostic model of survival for typical bronchial carcinoid tumours: analysis of 1109 patients on behalf of the European Association of Thoracic Surgeons (ESTS) Neuroendocrine Tumours Working Group. European Journal of Cardio-Thoracic Surgery 48 441–447. (10.1093/ejcts/ezu495) 25564217

[bib7] Fritz A Percy C Jack A Shanmugaratnam K & Sobin LH 2000 International Classification of Disease for Oncology, 3rd ed. Geneva, Switzerland: World Health Organization.

[bib8] García-Yuste M Matilla JM Cueto A Paniagua JM Ramos G Cañizares MA & Muguruza I 2007 Typical and atypical carcinoid tumours: analysis of the experience of the Spanish Multi-centric Study of Neuroendocrine Tumours of the Lung. European Journal of Cardio-Thoracic Surgery 31 192–197. (10.1016/j.ejcts.2006.11.031) 17196822

[bib9] Hallet J Law CH Karanicolas PJ Saskin R Liu N & Singh S 2015 Rural-urban disparities in incidence and outcomes of neuroendocrine tumors: A population-based analysis of 6271 cases. Cancer 121 2214–2221. (10.1002/cncr.29338) 25823667

[bib10] Humes KR Jones NA & Ramirez RR 2011 Overview of Race and Hispanic Origin: 2010 Census Briefs. Washington, DC, USA: United States Census Bureau. (available at: https://www.census.gov/content/dam/Census/library/publications/2011/dec/c2010br-02.pdf)

[bib11] Jemal A & Fedewa SA 2017 Lung cancer screening with low-dose computed tomography in the United States-2010 to 2015. JAMA Oncology 3 1278–1281. (10.1001/jamaoncol.2016.6416) 28152136 PMC5824282

[bib12] Kasajima A Konukiewitz B Schlitter AM Weichert W & Klöppel G 2022 An analysis of 130 neuroendocrine tumors G3 regarding prevalence, origin, metastasis, and diagnostic features. Virchows Archiv 480 359–368. (10.1007/s00428-021-03202-6) 34499237 PMC8986737

[bib13] Modlin IM Lye KD & Kidd M 2003 A 5-decade analysis of 13,715 carcinoid tumors. Cancer 97 934–959. (10.1002/cncr.11105) 12569593

[bib14] Oliveira AM Tazelaar HD Wentzlaff KA Kosugi NS Hai N Benson A Miller DL & Yang P 2001 Familial pulmonary carcinoid tumors. Cancer 91 2104–2109. (https://doi.org/10.1002/1097-0142(20010601)91:11<2104::aid-cncr1238>3.0.co;2-i) 11391591 10.1002/1097-0142(20010601)91:11<2104::aid-cncr1238>3.0.co;2-i

[bib15] Raz DJ Nelson RA Grannis FW & Kim JY 2015 Natural history of typical pulmonary carcinoid tumors: a comparison of nonsurgical and surgical treatment. Chest 147 1111–1117. (10.1378/chest.14-1960) 25539082 PMC4388119

[bib16] Rekhtman N 2010 Neuroendocrine tumors of the lung: an update. Archives of Pathology and Laboratory Medicine 134 1628–1638. (10.5858/2009-0583-RAR.1) 21043816

[bib17] Rekhtman N 2022 Lung neuroendocrine neoplasms: recent progress and persistent challenges. Modern Pathology 35(Supplement 1) 36–50. (10.1038/s41379-021-00943-2) 34663914 PMC8695375

[bib18] Samhouri BF Halfdanarson TR Koo CW McCarthy C Yi ES Thomas CF & Ryu JH 2023 DIPNECH: pragmatic approach, uncertainties, notable associations, and a proposal for an improved definition. Endocrine-Related Cancer 30 e230051. (10.1530/ERC-23-0051) 37410394

[bib19] Shah S Gosain R Groman A Gosain R Dasari A Halfdanarson TR & Mukherjee S 2021 Incidence and survival outcomes in patients with lung neuroendocrine neoplasms in the United States. Cancers (Basel) 13 1753. (10.3390/cancers13081753) 33916960 PMC8067543

[bib20] Steuer CE Behera M Kim S Chen Z Saba NF Pillai RN Owonikoko TK Khuri FR & Ramalingam SS 2015 Atypical carcinoid tumor of the lung: a surveillance, epidemiology, and end results database analysis. Journal of Thoracic Oncology 10 479–485. (10.1097/JTO.0000000000000419) 25371080

[bib21] Travis WD Rush W Flieder DB Falk R Fleming MV Gal AA & Koss MN 1998 Survival analysis of 200 pulmonary neuroendocrine tumors with clarification of criteria for atypical carcinoid and its separation from typical carcinoid. American Journal of Surgical Pathology 22 934–944. (10.1097/00000478-199808000-00003) 9706973

[bib22] Travis WD Brambilla E Nicholson AG Yatabe Y Austin JHM Beasley MB Chirieac LR Dacic S Duhig E Flieder DB, *et al.* 2015 The 2015 world health organization classification of lung tumors: impact of genetic, clinical and radiologic advances since the 2004 classification. Journal of Thoracic Oncology 10 1243–1260. (10.1097/JTO.0000000000000630) 26291008

[bib23] Yang J Schupp CW Harrati A Clarke C Keegan THM & Gomez SL 2014 Developing an Area-Based Socioeconomic Measure from American Community Survey Data. Fremont, CA, USA: Cancer Prevention Institute of California.

[bib24] Yost K Perkins C Cohen R Morris C & Wright W 2001 Socioeconomic status and breast cancer incidence in California for different race/ethnic groups. Cancer Causes and Control 12 703–711. (10.1023/a:1011240019516) 11562110

