# Supplementary material for: Survival outcomes for lung neuroendocrine tumors in California differ by sociodemographic factors
Source: Endocr Relat Cancer. 2023 Dec 8;31(1):e230068. doi: 10.1530/ERC-23-0068 (PMC10762535; doi:10.1530/ERC-23-0068)
Supplement: Supplementary Tables [file supplementary_tables.pdf]

SUPPLEMENTAL TABLE 1. Demographic and Clinical Characteristics of Lung NET Population by Histology

Variable	Level	Typical Carcinoid (n=5,569)	Atypical Carcinoid (n=469)	*p* value[a]
Age at diagnosis	Median [IQR]	64 [53, 73]	65 [54, 74]	**0.037**
Sex[b]	Male	1,672 (30.0%)	152 (32.4%)	0.54
	Female	3,896 (70.0%)	317 (67.6%)	
Diagnosis decade	1992-2000	1,297 (23.3%)	2 (0.4%)	**<0.001**
	2001-2009	1,668 (30.0%)	111 (23.7%)	
	2010-2019	2,604 (46.8%)	356 (75.9%)	
Race/Ethnicity[b]	NH White	4,097 (74.0%)	324 (69.4%)	**0.043**
	Hispanic	889 (16.0%)	78 (16.7%)	
	NH Black	276 (5.0%)	38 (8.1%)	
	Asian/Pacific Islander	250 (4.5%)	25 (5.4%)	
	American Indian	28 (0.5%)	2 (0.4%)	
County	Urban	3,999 (71.8%)	312 (66.5%)	**0.020**
	Suburban	1,429 (25.7%)	138 (29.4%)	
	Rural	141 (2.5%)	19 (4.0%)	
Marital status[b]	Unmarried	2,254 (41.8%)	182 (40.2%)	
	Married	3,140 (58.2%)	271 (59.8%)	0.50
Neighborhood SES at diagnosis[b]	Quintile 1 (lowest nSES)	618 (11.1%)	54 (11.1%)	0.78
	Quintile 2	953 (17.1%)	83 (17.7%)	
	Quintile 3	1,240 (22.3%)	114 (24.3%)	
	Quintile 4	1,341 (24.1%)	107 (22.8%)	
	Quintile 5 (highest nSES)	1,416 (25.4%)	111 (23.7%)	
Charlson comorbidities index[b]	None	2,447 (51.0%)	183 (46.6%)	0.22
	1-2	1,908 (39.8%)	168 (42.8%)	
	≥3	442 (9.2%)	41 (10.7%)	
Stage at diagnosis	Localized	3,797 (68.2%)	211 (45.0%)	**<0.001**
	Regional	943 (16.9%)	133 (28.4%)	
	Distant	627 (11.3%)	112 (23.9%)	
	Missing or unknown	202 (3.6%)	13 (2.8%)	
Tumor size (cm)	Median [IQR]	2.0 [1.3, 3.0]	2.5 [1.6, 4.2]	**<0.001**
Insurance[b,c]	Private only	2,593 (52.3%)	220 (47.6%)	0.11
	Medicare only, or Medicare + Private	1,656 (33.4%)	160 (34.6%)	
	Medicade/Military/Other Public	650 (13.1%)	77 (16.7%)	
	None/Self Pay	63 (1.3%)	5 (1.08%)	

Demographic and clinical characteristics of the study population by histology (typical versus atypical carcinoid). Data are presented as number of patients (%) unless otherwise indicated. [a]*p* value for difference between typical vs atypical carcinoid obtained from chi-square test for categorical variables or Wilcoxon rank sum test for age at diagnosis. Bolded values represented values that reach statistical significance. [b]Counts do not add up to 6,038 due to missing data. [c]Insurance payer reporting was not mandatory in the California Cancer Registry prior to 1996, so insurance data are presented for the N=5,527 cases diagnosed after 1995.

SUPPLEMENTAL TABLE 2. Sensitivity Analysis for Model 1, Association between Sociodemographic and Disease Factors with Overall Survival

		Model 1 – No Stage Covariate		Model 1	
**Variable**	**Level**	**HR [95% CI]**	***p* value**	**HR [95% CI]**	***p* value**
Sex	Male	1	-	1	-
	Female	0.60 [0.54, 0.65]	**<0.001**	0.59 [0.54, 0.65]	**<0.001**
Race/ethnicity	NH White	1	-	1	-
	Hispanic	0.97 [0.85, 1.11]	0.65	0.94 [0.82, 1.07]	0.34
	NH Black	1.38 [1.15, 1.65]	**0.001**	1.18 [0.98, 1.42]	0.073
	Asian/Pacific Islander	0.94 [0.75, 1.19]	0.63	0.90 [0.72, 1.13]	0.28
	American Indian	1.15 [0.68, 1.93]	0.60	1.33 [0.79, 2.22]	0.28
County	Urban	1	-	1	-
	Suburban	1.13 [1.03, 1.25]	**0.010**	1.12 [1.01, 1.23]	**0.024**
	Rural	0.77 [0.58, 1.02]	0.066	0.83 [0.63, 1.10]	0.19
Marital status	Unmarried	1	-	1	-
	Married	0.77 [0.70, 0.84]	**<0.001**	0.74 [0.68, 0.81]	**<0.001**
nSES	Quintile 1 (lowest nSES)	1	-	1	-
	Quintile 2	1.00 [0.86, 1.17]	0.99	1.00 [0.86, 1.18)	0.94
	Quintile 3	0.89 [0.77, 1.04]	0.14	0.92 [0.79, 1.07]	0.28
	Quintile 4	0.70 [0.60, 0.82]	**<0.001**	0.77 [0.66, 0.90]	**0.001**
	Quintile 5 (highest nSES)	0.65 [0.56, 0.76]	**<0.001**	0.72 [0.62, 0.84]	**<0.001**
Stage	Local			1	-
	Regional			1.50 [1.34, 1.69]	**<0.001**
	Distant			3.46 [3.09, 3.88]	**<0.001**
	Unknown			2.36 [1.96, 2.84]	**<0.001**
Diagnosis decade	1992-2000	1	-	1	-
	2001-2009	0.84 [0.76, 0.93]	**0.001**	0.77 [0.69, 0.85]	**<0.001**
	2010-2019	0.76 [0.67, 0.86]	**<0.001**	0.70 [0.62, 0.79]	**<0.001**

Multivariable Cox regression models of overall survival stratified by age. Model 1 was adjusted for sociodemographic and basic disease characteristics, including sex, race/ethnicity, county, marital status, nSES. stage, and decade of diagnosis. To better understand contribution of stage on associations between race/ethnicity and overall survival, we ran an additional Model 1 without the stage covariate. Abbreviations: HR, hazard ratio; CI, confidence interval; NH, non-Hispanic; nSES, neighborhood socioeconomic status

SUPPLEMENTAL TABLE 3. Sensitivity Analysis for Model 3, Addition of Comorbidities to Fully-Adjusted Overall Survival Model

		**Overall Survival Model 3**	
**Variable**	**Level**	**HR [95% CI]**	***p* value**
Sex	Male	1	-
	Female	0.64 [0.58, 0.70]	**<0.001**
Race/ethnicity	NH White	1	-
	Hispanic	0.89 [0.78, 1.02]	0.10
	NH Black	1.00 [0.83, 1.21]	0.97
	Asian/Pacific Islander	0.79 [0.63, 0.99]	**0.044**
	American Indian	1.39 [0.83, 2.23]	0.21
County	Urban	1	-
	Suburban	1.08 [0.98, 1.19]	0.12
	Rural	0.87 [0.66, 1.15]	0.34
Marital status	Unmarried	1	-
	Married	0.79 [0.72, 0.87]	**<0.001**
nSES	Quintile 1 (lowest nSES)	1	-
	Quintile 2	1.03 [0.88, 1.20]	0.72
	Quintile 3	0.99 [0.85, 1.16]	0.92
	Quintile 4	0.83 [0.71, 0.97]	**0.019**
	Quintile 5 (highest nSES)	0.80 [0.68, 0.93]	**0.005**
Stage	Local	1	-
	Regional	1.29 [1.15, 1.45]	**<0.001**
	Distant	2.11 [1.85, 2.40]	**<0.001**
	Unknown	1.46 [1.19, 1.78]	**<0.001**
Charlson comorbidities index	None	1	-
	1-2	1.45 [1.31, 1.60]	**<0.001**
	≥3	2.76 [2.37, 3.21]	**<0.001**
	Unknown	1.02 [0.88, 1.19]	0.78
Diagnosis decade	1992-2000	1	-
	2001-2009	0.68 [0.61, 0.76]	**<0.001**
	2010-2019	0.57 [0.50, 0.65]	**<0.001**
Histology	Typical carcinoid	1	-
	Atypical carcinoid	1.85 [1.57, 2.18]	**<0.001**
Treatment	Surgery (no)	1	-
	Surgery (yes)	0.47 [0.42, 0.53]	**<0.001**
	Radiation (no)	1	**-**
	Radiation (yes)	1.42 [1.20, 1.68]	**<0.001**
	Chemotherapy (no)	1	-
	Chemotherapy (yes)	1.96 [1.65, 2.32]	**<0.001**
	Chemotherapy (unknown)	1.67 [1.07, 2.61]	**0.023**
	Hormone treatment (no)	1	-
	Hormone treatment (yes)	0.71 [0.42, 1.19]	0.19
	Immune treatment (no)	1	-
	Immune treatment (yes)	1.71 [1.06, 2.27]	**0.027**

Multivariable Cox regression model of overall survival stratified by age. Model 3 was adjusted for sociodemographic and basic disease characteristics, including sex, race/ethnicity, county, marital status, nSES. stage, decade of diagnosis, histology, plus treatment variables. Here, Charlson comorbidity index is included as an additional covariate.
Abbreviations: HR: hazard ratio; CI, confidence interval; NH, non-Hispanic; nSES, neighborhood socioeconomic status

SUPPLEMENTAL TABLE 4. Sensitivity Analysis for Model 3, Addition of Comorbidities to Fully-Adjusted Lung Cancer-Specific Survival Model

		Cancer-Specific Survival Model 3	
**Variable**	**Level**	**SHR [95% CI]**	***p* value**
Age at diagnosis	<45	1	-
	45-54	1.84 [1.23, 2.74]	**0.003**
	55-65	2.62 [1.81, 3.79]	<**0.001**
	65-74	3.28 [2.28, 4.71]	<**0.001**
	≥75	3.91 [2.68, 5.71]	<**0.001**
Sex	Male	1	-
	Female	0.66 [0.56, 0.78]	**<0.001**
Race/ethnicity	NH White	1	-
	Hispanic	0.86 [0.67, 1.11]	0.24
	NH Black	1.07 [0.79, 1.44]	0.67
	Asian/Pacific Islander	0.87 [0.57, 1.34]	0.53
	American Indian	2.36 [1.18, 4.75]	**0.016**
County	Urban	1	-
	Suburban	1.11 [0.93, 1.33]	0.24
	Rural	0.79 [0.48, 1.30]	0.35
Marital status	Unmarried	1	-
	Married	0.77 [0.66, 0.91]	**0.002**
nSES	Quintile 1 (lowest nSES)	1	-
	Quintile 2	1.32 [0.98, 1.78]	0.072
	Quintile 3	1.32 [0.99, 1.76]	0.056
	Quintile 4	0.93 [0.69, 1.26]	0.64
	Quintile 5 (highest nSES)	0.96 [0.70, 1.32]	0.82
Stage	Local	1	-
	Regional	2.13 [1.74, 2.62]	**<0.001**
	Distant	3.37 [2.71, 4.20]	**<0.001**
	Unknown	1.75 [1.23, 2.50]	**0.002**
Charlson comorbidities index	None	1	-
	1-2	1.33 [1.12, 1.59]	**0.002**
	≥3	1.48 [1.10, 1.98]	**0.009**
	Unknown	1.07 [0.84, 1.37]	0.59
Diagnosis decade	1992-2000	1	-
	2001-2009	0.60 [0.50, 0.73]	**<0.001**
	2010-2019	0.38 [0.31, 0.48]	**<0.001**
Histology	Typical carcinoid	1	-
	Atypical carcinoid	2.77 [2.17, 3.55]	<**0.001**
Treatment	Surgery (no)	1	-
	Surgery (yes)	0.46 [0.38, 0.56]	**<0.001**
	Radiation (no)	1	-
	Radiation (yes)	1.78 [1.39, 2.29]	**<0.001**
	Chemotherapy (no)	1	-
	Chemotherapy (yes)	2.15 [1.66, 2.79]	**<0.001**
	Chemotherapy (unknown)	2.67 [1.45, 4.94]	**0.002**
	Hormone treatment (no)	1	-
	Hormone treatment (yes)	0.57 [0.26, 1.24]	0.16
	Immune treatment (no)	1	-
	Immune treatment (yes)	1.50 [0.85, 2.66]	0.17

Competing risks regression models of lung cancer specific survival with subdistribution hazard ratios obtained from the Fine-Gray Model. Model 3 was adjusted for sociodemographic and basic disease characteristics, including age, sex, race/ethnicity, county, marital status, nSES. stage, decade of diagnosis, histology, plus treatment variables. Here,

Charlson comorbidity index is included as an additional covariate. Abbreviations: SHR: subdistribution hazard ratio; CI, confidence interval; NH, non-Hispanic; nSES, neighborhood socioeconomic status
